# Phosphoric Acid Modified With Polyphenol-Rich Plant Extracts: Bond Strength to Sound and Eroded Dentin

**DOI:** 10.3290/j.jad.c_1951

**Published:** 2025-04-03

**Authors:** Thiago Saads Carvalho, Tommy Baumann, Mohamed Ahmed Said Zayed, Alessandro D. Loguercio, Anne Peutzfeldt, Samira Helena Niemeyer

**Affiliations:** a Thiago Saads Carvalho Associate Professor, Department of Restorative, Preventive and Pediatric Dentistry, University of Bern, Bern, Switzerland. Research idea and experimental design, funding acquisition, performed statistical analysis, contributed substantially to discussion and proofread the manuscript.; b Tommy Baumann Research Associate, Department of Restorative, Preventive and Pediatric Dentistry, University of Bern, Bern, Switzerland. Research idea and experimental design, funding acquisition, contributed substantially to discussion and proofread the manuscript.; c Mohamed Ahmed Said Zayed Doctoral Student, Department of Restorative, Preventive and Pediatric Dentistry, University of Bern, Bern, Switzerland. Performed the experiment and proofread the manuscript.; d Alessandro D. Loguercio Professor, Department of Restorative Dentistry, Ponta Grossa State University, Ponta Grossa, Paraná, Brazil. Contributed substantially to discussion and proofread the manuscript.; e Anne Peutzfeldt Research Associate, Department of Restorative, Preventive and Pediatric Dentistry, University of Bern, Bern, Switzerland. Contributed to experimental design, statistical analysis, contributed substantially to discussion and proofread the manuscript.; f Samira Helena Niemeyer Assistant Professor, Department of Restorative, Preventive and Pediatric Dentistry, University of Bern, Bern, Switzerland. Research idea and experimental design, funding acquisition, supervised the experiments, performed statistical analysis, created the figures, wrote original draft and proofread the manuscript.

**Keywords:** bond strength, cross-linkers, dental restoration, phosphoric acid, polyphenol, longevity, surface characterization

## Abstract

**Purpose:**

To modify phosphoric acid (PA) with polyphenol-rich plant extracts and verify their effect on immediate (24 h) and long-term (1 year) micro-shear bond strength (µSBS) of an adhesive system to sound and eroded dentin.

**Materials and Methods:**

420 dentin specimens (360 for µSBS and 60 for characterization) were prepared and divided into two substrate-subgroups: sound (untreated) and eroded dentin (underwent 10 cycles of 1 h exposure to human saliva and 5 min immersion in citric acid). The specimens from each subgroup were randomly distributed into six groups, according to PA (n = 30/group): PA-EXP (experimental control), PA-GSE (PA-EXP + grape seed extract), PA-BLU (PA-EXP + blueberry extract), PA-CRA (PA-EXP + cranberry extract), PA-GRE (PA-EXP + green tea extract), PA-COM (commercial control). After etching with the respective PA (15 s), specimens were restored with adhesive and composite resin. Half of the specimens of each group were subjected to µSBS testing after 24 h and the other half after 1 year of storage (tap water, 37°C). Analyses were made with three-way ANOVA and post-hoc Tukey tests (α = 0.05).

**Results:**

Higher µSBS was observed to sound dentin than to eroded dentin, regardless of the storage time, except for PA-BLU and PA-GSE after 1 year (p = 0.40 and p = 0.10, respectively). After 24 h, for both substrates, PA-COM presented statistically significantly lower µSBS than the other PAs. After 1 year, µSBS was significantly reduced for all groups except for the PA-COM (sound: p = 0.67; eroded: p = 0.13).

**Conclusion:**

Compared to the commercial PA, the modified PAs improved the immediate µSBS and gave similar long-term µSBS to sound as well to eroded dentin.

The essential, life-sustaining habit of eating and drinking can be harmful to our teeth if such habits are unbalanced and rich in fermentable carbohydrates or acidic substances. The constant exposure of our teeth to chemical and physical impacts can lead to several oral health problems, including dental caries and erosive tooth wear, which can quickly reach the dentin. When restoring lesions involving dentin, one must bear in mind the interplay between the collagenases (matrix metalloproteinases, MMPs) present in saliva and the dentin itself and the organic matrix of the dentin (rich in type I collagen).^19,24^ Acid attacks are common in erosive tooth wear lesions, and eroded dentin has shown lower bond strength, both immediately and in the long term.^15,45^ This may occur due to lower dentin hybridization, which, in turn, results in hydrolytic degradation of the hybrid layer.^14,40,45^ This is because the MMPs from saliva and dentin are activated during acid attacks, and they will degrade the organic matrix,^4,9,18,41^ considerably affecting the hybrid layer and composite resin restorations.^30^

Although the formation of an adequate hybrid layer is very important for the efficiency of adhesive systems for the eroded dentin, incomplete resin infiltration into the demineralized matrix occurs due to higher water content and inferior polymerization.^43^ This results in hydrolysis of the adhesive/resin system by esterases, as well as degradation of the collagen in the organic matrix by MMPs.^26,36^ Therefore, one way to improve the longevity of composite restorations could be to either use MMP inhibitors or dentin biomodifiers (cross-linking agents) during or after dentin etching.^26,31,35^ The latter has the ability to increase the mechanical properties of dentin collagen and to reduce collagen degradation.^5,11^ Another option to improve the longevity of restorations could be the longer and more active application of the primer to ensure the penetration of the monomers throughout the exposed collagen network.^37^

Plant extracts rich in polyphenols can act as both MMP inhibitors and cross-linking agents.^6,36^ Examples of such extracts are green tea, blueberry, and grape seed. These extracts can protect dentin against erosive challenges^33,34^ and reduce collagen degradation of dentin.^7, 8^ Some agents have already been tested either as additional primers after dentin etching or as ingredients in adhesives and primers.^1,3,26^ Contradicting results have been observed, especially when the agents were added to the adhesive, most likely because they impaired the polymerization.^27,44^

These agents, however, may instead be added to the phosphoric acid, and they can act on the collagen organic matrix during the etching step. To the present moment, only a handful of studies have evaluated the effect of phosphoric acids modified with natural agents,^13,21,25,29^ and even fewer studies have tested these acids on different dentin substrates, such as eroded dentin.^39^ Therefore, the aim of the present study was to modify the phosphoric acid with polyphenol-rich plant extracts and to verify their effect on the immediate and long-term micro-shear bond strength (µSBS) of a three-step etch-and-rinse adhesive system to sound and eroded dentin. We hypothesized that the modified phosphoric acids would create similar etch patterns on the dentin, but it would hinder degradation of the collagen organic matrix, and this would, in turn, improve not only the immediate bond strength of composite resin but especially the long-term bond strength to the dentin.

## MATERIAL AND METHODS

The present study was divided into three parts: 1) Modification of the phosphoric acids (PAs); 2) Micro-shear bond strength (µSBS) of composite restorations performed with the PAs to sound and eroded dentin; and 3) Characterization of the dentin etch patterns.

### Modification of the Phosphoric Acid (PA)

A (37%) solution of phosphoric acid (PA) was prepared in the laboratory and, from that, a total of four modified phosphoric acid groups (Table 1) were developed containing polyphenols, and an experimental control group was left without polyphenols. The modified groups were prepared by adding a polyphenol-rich plant extract solution to obtain a final concentration of 2% polyphenol and 37% phosphoric acid, according to the respective plant extract (Table 1): grape seed extract (GSE); blueberry extract (BLU); cranberry extract (CRA); and green tea extract (GRE). The experimental control and the four modified groups were then gelled by adding 6 g of polyethylene glycol (as used in some commercialized acids). Additionally, a commercial control (COM; the phosphoric acid Gel Etchant from Kerr, Brea, CT, USA) was used (Table 1).

**Table 1:** Description of experimental groups

Name	Group	Ingredients
PA-EXP	Experimental control	Phosphoric acid solution (37%), 0.6 g/ml polyethylene glycol	
PA-GSE	Grape seed extract	2% polyphenols from grape seed extract
PA-BLU	Blueberry extract	2% polyphenols from blueberry extract
PA-CRA	Cranberry extract	2% polyphenols from cranberry extract
PA-GRE	Green tea extract	2% polyphenols from green tea extract
PA-COM	Commercial control	Phosphoric acid gel (37.5%)
Blueberry extract (Heidelbeer-Extrakt mit Anthocyanen, Fairvital B.V., Germany), green tea extract (Green Tea Deluxe, ZeinPharma, Germany), and grape seed extract (OPC, Fairvital B.V., Germany), cranberry extract (BioProphyl®, Urocyan, Germany), Gel Etchant, Kerr, Brea, CT, USA.

The pH of the solutions and gels were analyzed with pH indicator strips (pH 0-14, MQuant, Merck, Darmstadt, Germany).

### Preparation of Dentin Specimens

A total of 420 dentin specimens (360 for µSBS analyses and 60 for characterization of the dentin etch patterns, Fig 1) were prepared from sound-extracted permanent human molars, obtained from a pooled bio-bank. The teeth were previously extracted by dental practitioners in Switzerland. Before the extraction, the patients were informed about the use of their teeth for research purposes and their consent was obtained. The local ethical committee considers pooled bio-banks as irreversibly anonymized, and they waive the necessity of previous ethical approval. The present experiment was carried out in accordance with the approved guidelines and regulations of the local ethical committee (Kantonale Ethikkommission).

The occlusal surfaces of the teeth were ground with 220-grit and 500-grit silicon carbide abrasive papers (LaboPol-21, Struers, Ballerup, Denmark), under constant water cooling, until the mid-coronal dentin was exposed. Then, the roots were shortened using a water-cooled diamond saw (IsoMet Low Speed Saw, Buehler, Lake Bluff, IL, USA) and embedded in self-curing acrylic resin (Paladur, Heraeus Kulzer, Hanau, Germany). The specimens were rinsed with deionized water in an ultrasound bath (60 s). Half of the specimens (n = 210) were kept in a humid chamber at 4ºC, until the beginning of the experiment (sound dentin specimens), and the other half (n = 210) underwent erosive challenges (eroded dentin).

Prior to the erosive challenge, a standardized smear layer was formed on the dentin specimens by grinding for 5 s on 500-grit silicon carbide abrasive paper (LaboPol-21, Struers, Ballerup, Denmark), under water cooling. The specimens were then submitted to 10 erosive cycles, each consisting of 1 h exposure to human saliva (37ºC), followed by 5 min immersion in citric acid (1%, pH 3.6, 25ºC). Human saliva was used because it contains MMPs, influencing the degradation of the organic matrix. Whole mouth stimulated saliva samples were collected from fifteen volunteers, pooled, centrifugated, and the supernatant was frozen until use. Again, the local ethical committee considers pooled saliva as irreversibly anonymized samples, and prior ethical approval is not required. Once the 10 cycles were over, the specimens were kept in a humid chamber at 4ºC until the time of experiment.^33,42^

### Micro-shear Bond Strength (µSBS)

#### Experimental procedure

The 360 dentin specimens prepared for µSBS (180 sound and 180 eroded) were used in this part of the study. Half of the specimens from each subgroup were used to determine the immediate (24 h) µSBS, while the other half was used to determine the long-term (1 year) µSBS (Fig 1a).

All specimens were placed in tap water and kept in room temperature for one hour prior to the experiment. On the eroded dentin specimens, a standardized smear layer had been created prior to erosion, as described above, and the specimens were ready for bonding and restoration. On the sound dentin specimens, the smear layer was created (by grinding for 5 s on 500-grit silicon carbide abrasive paper; LaboPol-21, Struers, Ballerup, Denmark) immediately before bonding and restoration.

For bonding and restoration, the dentin specimens were briefly water-sprayed and gently air-dried. A self-adhesive tape with a perforation in the center (~2 mm ∅) was placed on the dentin surfaces to guarantee a defined, isolated, and standardized bonding area. The dentin specimens were then subdivided into the different PA groups (Table 1), with a total of n = 30 sound and n = 30 eroded specimens per group. The specimens were etched with the respective PA (Table 1): 15 s etching, then 15 s rinsing with water spray and 5 s slightly air-dried. Subsequently, the adhesive system (OptiBond FL, Kerr, Orange, CA, USA) was applied according to the manufacturer’s instructions. First, the OptiBond FL Primer was applied with a microbrush (OminiDent, Ominibrush Eco Blau, Nieder-Roden, Germany) lightly scrubbed for 15 s, and gently air-dried for 5 s. Next, the OptiBond FL Adhesive was applied also with a microbrush, creating a thin coating and light cured for 10 s (LED curing unit). For the restorative procedure, a split Teflon mold was fixed on the dentin surface (inner diameter 1.5 mm, bonding area of 1.8 mm^2^, height 2 mm) and filled with composite resin (Filtek Z250, Solventum, St. Paul, MN, USA; shade A4). The composite was light cured for 20 s (LED curing unit). The specimens were immediately placed in black light-tight boxes to avoid any further influence of ambient light. All specimens were kept in light-tight boxes and in an incubator (Memmert UM 500, Memmert & Co., Schwabach, Germany) at 37°C and 100% humidity. All light-curing was performed with the same LED curing unit (Demi LED, Kerr Corporation, Middleton, WI, USA); and the light power density was verified before the experiment by a radiometer (Bluephase Meter, Ivoclar Vivadent, Liechtenstein) to be at least 1500 mW/cm^2^.

After 24 h, half of the specimens of each PA group (n = 15 sound and n = 15 eroded specimens) were subjected to µSBS testing. The other half of the specimens (n = 15 sound and n = 15 eroded specimens/PA) were transferred to light-tight boxes filled with tap water and stored for 1 year at 37°C. The water was renewed monthly.

#### µSBS analysis

After 24 h or 1 year, µSBS were determined with a universal testing machine (Zwick Z1.0 TN, Zwick), using a stainless-steel wire (diameter 0.6 mm) at a crosshead speed of 1 mm/min, as previously described.^32,38^ The maximum force (N) was recorded and the µSBS (in MPa) was calculated: maximum force (N) divided by the bonding area (1.8 mm^2^). The failure mode of each specimen was determined under a magnifying glass (2.5×), and classified as: 1) cohesive failure in dentin; 2) adhesive failure at the dentin–adhesive interface; 3) adhesive failure at adhesive–composite resin interface; 4) cohesive failure in composite; or 5) mixed adhesive failure (combinations of failure modes 1 to 4).

#### Statistical analysis

The µSBS data were analyzed with Q-Q plots, Shapiro-Wilk and Levene’s tests for normality and equality of variances. Three-way ANOVA was used to evaluate µSBS values, considering the factors “substrate” (in two levels: sound and eroded dentin), “PA” (in six levels: PA-EXP, PA-GSE, PA-BLU, PA-CRA, PA-GRE, PA-COM) and “storage time” (in two levels: 24 h and 1 year). Differences between the groups were analyzed using Tukey post-hoc tests. Analyses were performed with IBM SPSS Statistical tool (v23), and the significance level was set at 5%. Failure modes are shown in percentage per group. Also, χ2 tests were performed using OpenEpi (v3) to verify the differences in the types of failure according to the incubation time, separately for sound and eroded dentin.

### Characterization of the Dentin Etch Pattern and Collagen Layer Created by the PAs

The remaining 60 dentin specimens (n = 30 sound and n = 30 eroded specimens) were used for profilometric, roughness, and microscopic (scanning electron microscopy – SEM) characterization of the etch pattern and collagen layer. Each specimen was sectioned in the middle into two parts: one part was used for profilometric, roughness, and microscopic (SEM) characterization of the etch pattern, and the other part was used for the characterization of the organic matrix layer (Fig 1b).

For the profilometric, roughness, and microscopic characterization, an area of 2 × 1 mm was scanned with an optical profilometer (MicroProf 100, FRT the art of metrology, Kirchheim, Germany) in the middle of the dentin specimen. This initial scan provided the initial curvature. Then, three lines with a higher resolution were scanned to obtain information on surface roughness (Ra) of each dentin specimen. After the initial analysis, half of the surface of each specimen was protected with an adhesive tape to preserve an intact (reference) area that was not subjected to PA, while the other half was subjected to acid etching with the respective PA (n = 5 sound and n = 5 eroded specimens/PA) as described above. Profilometric analyses were performed to assess the demineralization depth caused by the PAs. For that, the adhesive tapes were removed, and the dentin surface was scanned again with the optical profilometer. Demineralization depth was determined by subtracting the mean height of the etched area from the mean height of the reference area. Surface roughness was determined by analyzing three lines of the scan again and comparing this result to the initial reference area. One representative specimen per group (of each PA and each sound/eroded subgroups) were directly mounted on aluminum stubs, sputter-coated with gold palladium and a sputter-coating device (100 s, 50 mA; Balzers SCD 050; Balzers, Liechtenstein), and the characterization of the etch pattern was descriptively analyzed using SEM (JSM-6010PLUS/LV SEM, JEOL, Tokyo, Japan).^22^

For the characterization of the organic matrix layer, the other part of the specimens was submitted to enzymatic degradation. They were incubated in a mineral solution containing collagenase from Clostridium histolyticum type VII (96 h, 37 °C), which should degrade the demineralized collagen layer in the dentin specimens.^34^ Subsequently, the dentin specimens underwent profilometric and roughness analyses (as described previously) to calculate the height of demineralized and subsequently degraded collagen layer. Likewise, one representative specimen (from each PA and each eroded/sound subgroups) was also used for the characterization of the dentin surface after organic matrix removal using the SEM (JSM-6010PLUS/LV SEM, JEOL, Tokyo, Japan).^17^

This part was performed as characterization of the dentin surface, so no statistical analysis was performed. Only descriptive results are presented.

## RESULTS

### Modification of the Phosphoric Acids (PAs)

The five experimental PAs were prepared successfully. The addition of polyethylene glycol resulted in a gel consistency that allowed the gels to stay on the dentin surface during the application time, very similar to the commercial gel. The commercial PA used (Gel Etchant, Kerr, Orange, CA, USA) was the one recommended by the manufacturer of the adhesive system used in the present experiment (OptiBond FL, Kerr, Orange, CA, USA). The addition of plant extracts and polyethylene glycol did not alter the pH. The pH of all groups, including the PA-COM, was close to 1 (data not shown).

### Micro-shear Bond Strength (µSBS)

**Fig 2:**
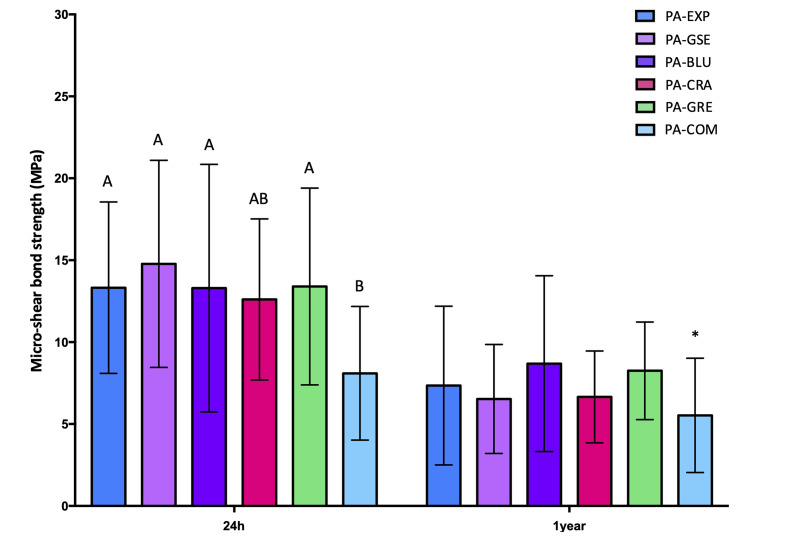
Mean and standard deviation µSBS for sound dentin specimens after 24 h and 1 year of storage. Different uppercase letters denote significant differences between the experimental groups after 24 h storage. No significant difference was observed between the groups after 1 year. *No significant difference was observed for the commercial control (PA-COM) between 24 h and 1 year (p = 0.68).

**Fig 3:**
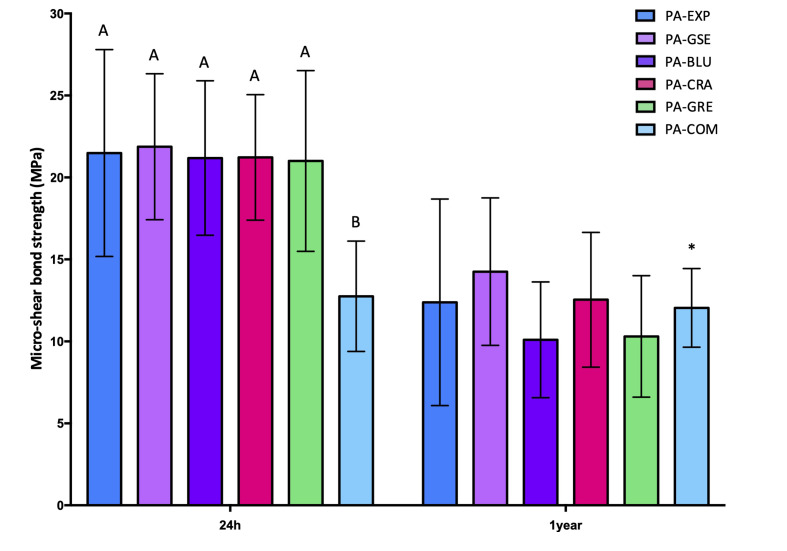
Mean and standard deviation µSBS for eroded dentin specimens after 24 h and 1 year of storage. Different uppercase letters denote significant differences between the six groups after 24 h storage. No significant difference was observed between the six groups after 1 year. *No significant difference was observed in groups PA-COM between 24 h and 1 year (p = 0.13).

The immediate (24 h) and long-term (1 year) µSBS results for the sound specimens are shown in Figure 2, and those for the eroded specimens in Figure 3. The results of failure mode analysis are reported in Tables 2 and 3 for sound and eroded dentin, respectively.

**Table 2:** Percentage of failure modes per group, for sound specimens

Time	Groups	Non-eroded specimwens
Failure mode 1	Failure mode 2	Failure mode 3	Failure mode 4	Failure mode 5
**24 h**	**PA-EXP**	0	0	0	0	100
**PA-GSE**	0	0	0	0	100
**PA-BLU**	0	7	0	0	93
**PA-CRA**	0	0	0	0	100
**PA-GRE**	0	7	0	0	93
**PA-COM**	0	0	0	0	100
**1 year**	**PA-EXP**	0	7	20	0	73
**PA-GSE**	0	0	20	0	80
**PA-BLU**	0	7	7	0	86
**PA-CRA**	0	7	20	0	73
**PA-GRE**	0	0	13	0	87
**PA-COM**	0	13	20	0	67
Failure mode 1: cohesive in dentinFailure mode 2: adhesive at the dentin surfaceFailure mode 3: adhesive between adhesive system and composite resin Failure mode 4: cohesive in composite resinFailure mode 5: mixed (mixed-type fracture pattern 1 to 4)

All three factors (substrate, PA, and storage time) were statistically significant (p <0.05). Also, the interactions between “PA” and “storage time” and between “substrate” and “storage time” were statistically significant (p <0.01 and p = 0.01, respectively). However, the interactions between “substrate” and “PA” (p = 0.54) and between all three factors (p = 0.15) were not statistically significant. Regardless of time point, the µSBS values were higher for the sound specimens than for the eroded ones, except for PA-BLU und PA-GRE after 1 year (p = 0.40 and p = 0.10, respectively).

For the sound specimens, after 24 h, all experimental PAs showed statistically significantly higher µSBS than the commercial control (p <0.01), with no differences between the experimental PAs (p >0.05). After 1 year, the µSBS had significantly decreased for the experimental PAs, but not for the commercial control (p = 0.67), so that the experimental PAs were still as effective as the PA-Comm.

For the eroded specimens, after 24 h, the commercial control presented lower µSBS than the experimental PAs, except PA-CRA (p = 0.13). No differences were observed among the experimental PAs. After 1 year, the µSBS had significantly decreased for the experimental PAs, but not for the commercial control (p = 0.13), so that the experimental PAs were still as effective as the PA-COM.

Tables 2 and 3 present the distribution of the failure modes after µSBS testing for sound and eroded specimens, respectively, considering each time point (24 h and 1 year). In general, the most frequent failure mode observed was mixed failure (failure mode 5) consisting of adhesive failure at the dentin surface and adhesive failure at the adhesive system – composite resin interface. Failure between the adhesive system and composite resin (failure mode 3) was mostly observed on sound dentin (Table 2), especially after 1 year (χ2 = 36.67; df = 2; p < 0.01), whereas pure adhesive failure at the dentin surface (failure mode 2) was most frequently observed on eroded dentin (Table 3), both after 24 h (χ2 = 33.56; df = 1; p < 0.01) and 1 year (χ2 = 17.67; df = 2; p <0.01).

**Table 3:** Percentage of failure modes per group, for eroded specimens

Time	Groups	Eroded specimens
Failure mode 1	Failure mode 2	Failure mode 3	Failure mode 4	Failure mode 5
**24 h**	**PA-EXP**	0	13	0	0	87
**PA-GSE**	0	7	0	0	93
**PA-BLU**	0	47	0	0	53
**PA-CRA**	0	27	0	0	73
**PA-GRE**	0	33	0	0	67
**PA-COM**	0	7	0	0	93
**1 year**	**PA-EXP**	0	20	0	0	80
**PA-GSE**	0	33	13	0	53
**PA-BLU**	0	13	7	0	80
**PA-CRA**	0	7	20	0	73
**PA-GRE**	0	7	20	0	73
**PA-COM**	0	40	7	0	53
Failure mode 1: cohesive in dentinFailure mode 2: adhesive at the dentin surfaceFailure mode 3: adhesive between adhesive system and composite resin Failure mode 4: cohesive in composite resinFailure mode 5: mixed (mixed-type fracture pattern 1 to 4)

For the sound specimens (Table 2), the most frequent failure mode was mixed failure (failure mode 5). Only PA-BLU and PA-GRE had 7% each of pure adhesive failure at the dentin surface (failure mode 2) after 24 h, and all groups presented more adhesive failure between the adhesive system and composite resin (failure mode 3) after 1 year.

Likewise, for the eroded specimens (Table 3), the most frequent failure mode was mixed failure (failure mode 5), but all the number of adhesive failures at the dentin surface (failure mode 2) was greater after 24 h and after 1 year. After 1 year, only groups PA-CRA and PA-GRE presented more adhesive failures between the adhesive system and composite resin (failure mode 3).

### Characterization of the Dentin Etch Pattern and Collagen Layer Created By the PAs

**Fig 4:**
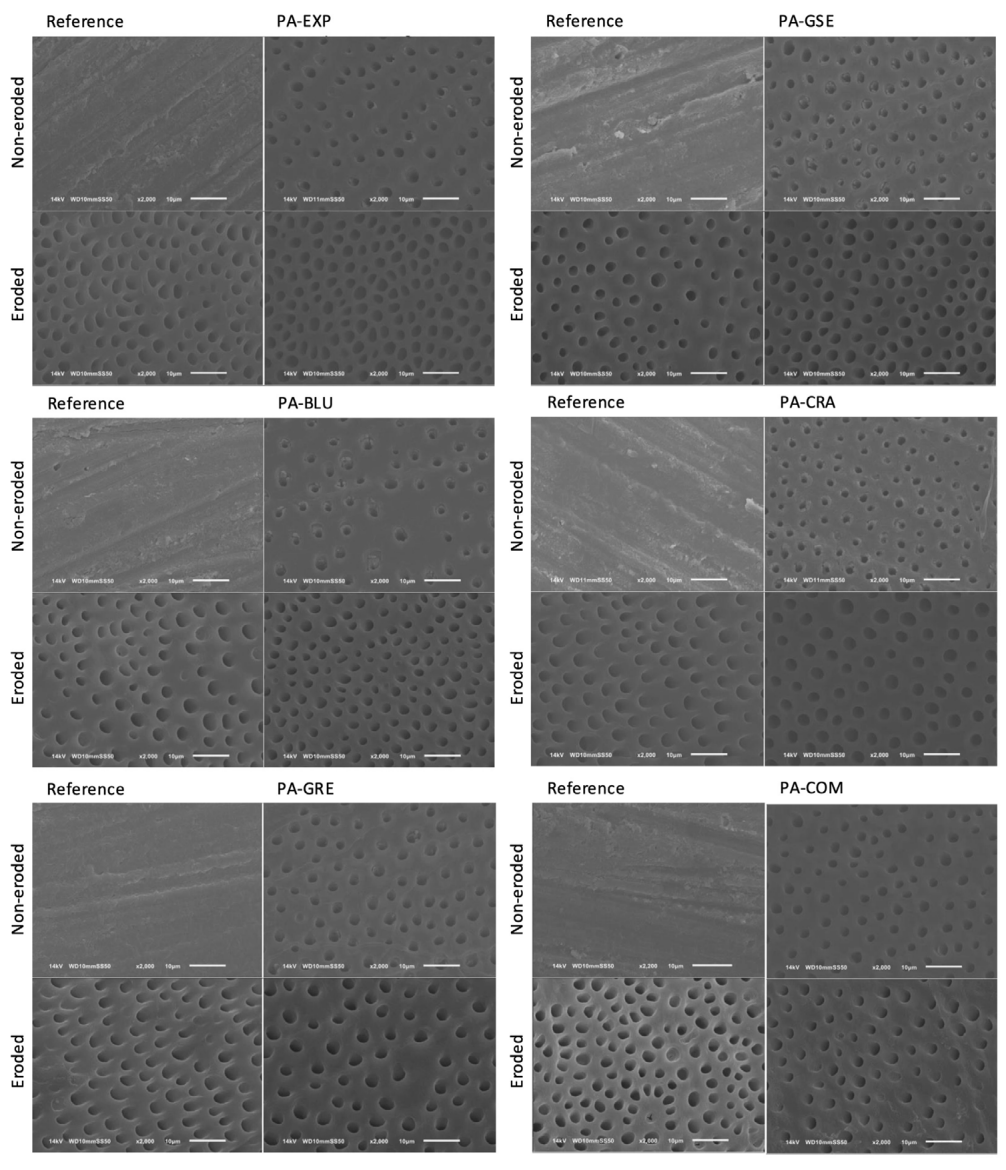
Scanning electron microscopy of the etch pattern of eroded and sound specimens after PA application. For each PA group, the left image represents the reference surfaces, and the right image represents the etched surfaces.

**Fig 5:**
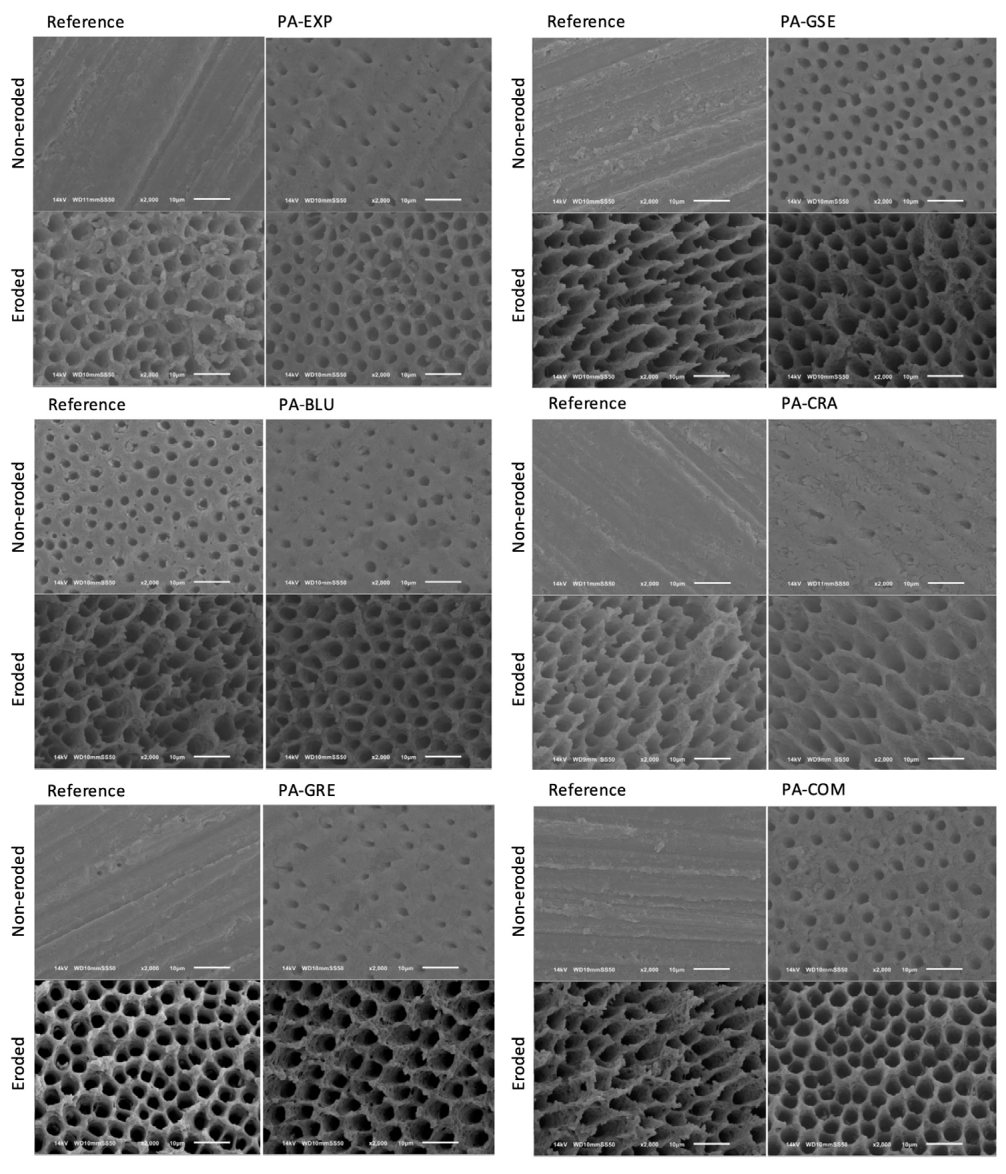
Scanning electron microscopy of the etch pattern of sound and eroded specimens after PA application and removal of demineralized collagen. For each PA group, the left image represents the reference surfaces (without PA), and the right image represents the etched surfaces after collagen removal.

Figures 4 and 5 show representative SEM images of the sound and eroded specimens for each group after application of PA (Fig 4) and after application of PA and immersion in a mineral solution containing collagenase for removal of the demineralized collagen (Fig 5). In both cases, the eroded specimens showed open tubules already on the reference surfaces. The type of PA did not seem to influence the etch pattern of the dentin specimens.

The immersion in a mineral solution containing collagenase influenced the surface characteristics mostly of the eroded specimens, but the type of PA did not influence the appearance of the dentin surfaces (Fig 5), being similar for all groups.

Tables 4 and 5 present the results of dentin surface loss after 15 s application of PA, thickness of collagen layer, and surface roughness for sound and eroded specimens, respectively. Due to the low number of specimens, the results are only descriptively presented.

**Table 4:** Dentin surface loss, surface roughness of the reference area (sound surface/standardized smear layer) and of the etched area (non-eroded surface/standardized smear layer and PA application) with and without removal of demineralized collagen. Thickness of degraded collagen layer and surface roughness after collagen removal (roughness collagen)

	Sound specimen
Groups	Surface loss (µm)	Collagen layer (µm)	Roughness
Reference (Ra)	After etched (Ra)	Collagen (Ra)
**Phosphoric acid application**	**PA-EXP**	0.03 (5.40)	–	0.53 (0.27)	0.66 (0.16)	–
**PA-GSE**	–1.09 (7.19)	–	0.65 (0.07)	0.82 (0.07)	–
**PA-BLU**	0.17 (5.99)	–	0.55 (0.19)	0.63 (0.15)	–
**PA-CRA**	–0.06 (3.97)	–	0.60 (0.06)	0.67 (0.09)	–
**PA-GRE**	3.60 (5.83)	–	0.70 (0.21)	0.73 (0.10)	–
**PA-COM**	–4.88 (7.19)	–	0.66 (0.16)	0.78 (0.18)	–
	Groups	Surface loss (µm)	Collagen layer (µm)	Roughness
Reference (Ra)	After etched (Ra)	Collagen (Ra)
**Phosphoric acid application and collagen removal**	**PA-EXP**	–6.82 (8.94)	7.74 (6.34)	0.57 (0.19)	0.78 (0.17)	0.69 (0.18)
**PA-GSE**	4.38 (5.53)	–8.17 (4.66)	0.74 (0.17)	0.85 (0.17)	0.77 (0.17)
**PA-BLU**	–5.53 (8.93)	5.20 (9.76)	0.70 (0.20)	0.78 (0.13)	0.60 (0.12)
**PA-CRA**	–4.67 (6.44)	0.21 (6.71)	0.59 (0.15)	0.75 (0.17)	0.71 (0.12)
**PA-GRE**	–2.66 (7.26)	2.57 (8.09)	0.58 (0.10)	0.86 (0.14)	0.68 (0.09)
**PA-COM**	–3.34 (4.14)	–1.16 (3.74)	0.65 (0.13)	0.76 (0.07)	0.77 (0.26)


In general, surface loss and thickness of the collagen layer varied between the groups, regardless of the substrate. For the sound specimens, the application of PA seems to have increased the surface roughness for all groups, but collagen removal slightly reduced the surface roughness for the experimental control and the PA-GSE, PA-BLU, and PA-GRE extracts. For the eroded specimens, no change in roughness was observed after application of the PAs. However, after collagen removal, the roughness increased for all groups.

## DISCUSSION

The present study analyzed three factors on micro-shear bond strength (µSBS) of an etch-and-rinse adhesive system: i) modified PA with four different polyphenol-rich plant extracts (grape seed, blueberry, cranberry, and green tea); ii) storage time at 24 h and 1 year; and iii) sound and eroded dentin. Results, in general, demonstrated that the three factors individually influenced the bond strength, but when the factors were analyzed together, their combined effect did not statistically significantly influence µSBS. Interestingly, the interactions between “PA” and “storage time,” or that between “substrate” and “storage time” were statistically significant. This means that µSBS was significantly higher for the sound specimens than for the eroded ones, both at 24 h and 1 year; and the modified PAs statistically significantly influenced µSBS, generally showing significantly higher µSBS than the commercial PA after 24 hour of water storage.

The higher µSBS to the sound specimens than to the eroded ones corroborates numerous previous reports^43^ and has been explained as follows: dental erosion leads to structural changes, like mineral loss and collagen exposure, resulting in the formation of a heterogeneous hybrid layer. This compromised layer, along with reduced resin infiltration due to high water content and inferior polymerization, contributes to lower bond strength values.^12,14,40,45^ Additionally, decreased calcium and phosphorus content in eroded dentin further impairs chemical bonding to dentin, exacerbating the reduction in bond strength.^46^

The morphological and physiological homogeneity of dentin has been emphasized as crucial for achieving uniform and reliable bonding.^10^ Considering that eroded dentin lacks these properties, partial removal of the eroded dentin with a diamond bur has been recommended.^45^ This leads to the removal of the non-uniform demineralized layer, exposing fresh sound dentin, which is more appropriate for bonding, with positive results were observed when compared to non-prepared eroded dentin.^45^ In contrast, the present study used eroded dentin without any bur preparation, as our aim was to test the modified PAs on the bonding to eroded dentin without the need for tooth substance removal. The PAs were modified with polyphenol-rich plant extracts, enabling dentin biomodification during the etching process.^13,21,25^ However, the modified PAs could not improve the bond strength to eroded dentin to the level of sound dentin, though they generally did improve the short-term (24 h) µSBS.

To help understand the interaction of the PAs with the dentin, particularly the organic matrix, the etch pattern and the exposed organic layer of the dentin were characterized. We had expected less change of the dentin organic layer (after collagenase) for the groups treated with the PAs containing plant extracts, since the polyphenols can act as cross-linkers, increasing the mechanical properties of the collagen layer,^5,11^ and thus protecting the dentin collagen against degradation.^7,28^ In contrast, we observed that the type of PA does not seem to influence the conditioning pattern of the dentin, regardless of the dentin condition. This highlights that the modification of the PA with plant extracts does not impair their effect, and that may explain why we found high values of µSBS. However, the collagenase degraded the organic layer and influenced the surface characteristics of the eroded specimens, which showed more open tubules and higher surface roughness. For the sound dentin specimens, the experimental PAs even showed reduced surface roughness after collagen removal. The reason for these different characteristics and their impact on the bond strength still need further investigation.

After 24 h storage, all experimental PAs showed statistically significantly higher µSBS than the commercial PA to both sound and eroded dentin in agreement with previous studies.^21,29,39^ However, after 1 year, the µSBS of the experimental PAs had been reduced and resulted in µSBSs similar to that of the commercial PA. The fact that, when using the modified PAs, we found µSBS values to have decreased after 1 year of storage was unexpected. One possible explanation is that the polyphenols, as used in this model, were not able to adequately cross-link the collagen, so any improvement of the hybrid layer was limited. This lack of strengthening of the hybrid layer in the long term seems to be reflected in the failure modes, in that there was no overall change in the failure modes observed after 24 h and 1 year for eroded specimens.

The concentration of polyphenols used to modify the PAs was based on earlier studies that investigated the ability of solutions containing 2% polyphenol from plant extracts to protect against dentin erosion.^33,34^ Moreover, this concentration is also in the range of concentrations of cross-linking agents tested previously in different materials (phosphoric acids, primers, bonds).^13,21,39^ Differences in the results are challenging to explain. A closer examination of the literature reveals that proanthocyanidin, particularly found in grape seed extracts, has been the most extensively studied polyphenol. It has been shown that 2% of proanthocyanidin was able to stabilize collagen and protect it against collagenase degradation.^28^ However, controversial results have been reported when grape seed extracts were evaluated in different studies. In two studies, the application of phosphoric acid containing grape seed extracts resulted in stable µTBS values after 6 and 12 months of water storage compared with commercial phosphoric acid.^21,29^ Conversely, another study observed lower µTBS values after thermocycling when dentin was conditioned with phosphoric acid containing GSEs.^13^ Variations in the concentration of proanthocyanidins, application times, and aging methods could explain these discrepancies. For example, while Hass et al^21^ and Loguercio et al^29^ used 10% proanthocyanidin applied for 30 s, De-Paula et al^13^ used 37% proanthocyanidin applied for 15 s. Additionally, the former studies stored specimens in water for 6 and 12 months, whereas the latter subjected the specimens to thermocycling.

However, all previous studies have focused on the application of PA modified with polyphenol-rich plant extracts on sound dentin, which is a different substrate from eroded dentin. The only study that evaluated the application of different modifications was conducted by Siqueira et al.^39^ In this study, PA modified with proanthocyanidin, and chlorhexidine was compared to commercial PA, and better results were observed for the former. However, only immediate bond strength results were reported. This underscores the importance of the present study, which is the first to report long-term (1 year) µSBS results for PA modified with polyphenol-rich plant extracts on eroded dentin. Unfortunately, in our study we did not observe any improvement in µSBS when using the modified PAs, as the µSBS decreased significantly after 1 year of storage for all experimental PAs. However, the fact that the modified PAs resulted in µSBS values comparable to those of the commercial PA, lends promise and encourages further investigations.

The commercial PA used in the present study is the one produced and recommended by the manufacturer of the adhesive system, OptiBond FL, itself. This adhesive system is considered the gold standard, as it has shown superior results in *in vitro* as well as in long-term clinical studies.^38^ Moreover, our research group has already performed other studies with the same commercial products and methods,^15,16,38^ and the present µSBS values with both the gold standard and the commercial PA in both sound and eroded dentin are in accordance with previous studies.^15,16,38^ Notwithstanding this similarity between the present and previous µSBS values, the present experimental PAs presented statistically significantly higher µSBS values than the commercial PA, though the reasons for these results are still not clear. However, these results can be associated with improvement of collagen through polyphenol cross-linking present in the experimental PAs.^13,20^

Remarkably, we did not observe any differences between the four modified PAs, using four different plant extracts. Although these different plant extracts have shown an effect against enzymatic degradation of the collagen, the type of polyphenol can impact the results, so differences in our results were also expected.^2^ Grape seed extract contains oligomers, resulting in more hydroxyl groups available for binding to the collagen matrix. The green tea extract has a high amount of catechins, especially epigallocatechin-3-gallate (EGCG), which has been shown to reduce collagen degradation.^2,6,23^ These effects would impact the stabilization of the dentin organic matrix, importantly influencing the dentin-resin interface durability and dentin demineralization processes.^2^ The reason for the lack of differences in the present study is unclear. Nevertheless, most of the studies modifying the PAs did not test the acids in gel form, as we did in the present study. It may be hypothesized that having the active ingredients in gel form may have impaired their availability and effect on the collagen layer. It is noteworthy that after adding the thickening agent (polyethylene glycol) to jellify the solutions, the final volume increased, which also affected the final concentration of the PA (around 25%) and of the polyphenols, an aspect which may be considered a limitation of the study. Still, this happened to all groups, and the polyphenol concentrations remained similar for all experimental groups. Furthermore, one cannot exclude the possibility that the polyethylene glycol may have impaired the effect of the polyphenols.

Other studies have added the polyphenols to primers or adhesives, but one advantage of including the polyphenol-rich plant extracts in the PA instead is the possibility of bringing the collagen cross-linking agents to the entire depth of dentin. While the acid is demineralizing the dentin, it was hypothesized that the polyphenols would also penetrate into the deeper layers. In other words, we may have a better distribution of the collagen cross-linking agents throughout the demineralized dentin. So, in case of incomplete infiltration of the resin monomers, polyphenols would be present, to some extent, which would inhibit the MMPs and reduce collagen degradation beneath and within the hybrid layer in the long term. Furthermore, these agents may cross-link the collagen, improving its mechanical properties and, consequently, the resin–dentin bond. Moreover, by adding the plant extracts to the PA, we not only avoid increasing the number of application steps in the adhesive system but also any negative influence on polymerization.^27^ However, we cannot exclude the fact that the high molecular weight of the polyphenols compared to the phosphoric acid (>600 Da versus 100 Da, respectively) might have impaired the permeation of the molecules deep into the collagen.^1^

In conclusion, the modified phosphoric acids, the storage time, and the erosion of dentin all influenced bond strength. Compared to the commercial phosphoric acid, the acids modified with plant extract improved the immediate (24 h) µSBS and gave similar long-term µSBS to non-eroded as well as to eroded dentin. Moreover, the modification of the phosphoric acids with the plant extracts did not impair the etch pattern of the dentin.

### Clinical Relevance

The addition of polyphenol-rich plant extracts to phosphoric acid is a promising method for improving dentin properties and dentin-resin bonding, without the impairment of the effect of phosphoric acid and without including extra application steps in the adhesive system.

### Acknowledgments

The authors would like to thank PD Dr. S. Flury(^†^) for helping conceive the study design, as well as I. Hug and M. Stiebritz for helping with the experimental procedures. This study was supported/funded by a grant from the German Society of Restorative and Regenerative Dentistry (DGR^²^Z) and GC. Part of the results were used in the doctoral thesis of the third author.

#### Declaration of competing interest

The authors report no potential conflict of interest.

#### Availability of data and materials

All data and materials used and/or analyzed during the current study are available from the corresponding author upon reasonable request.

**Fig 1a and b:** Flowchart of the experimental procedures. (a) Dentin specimens and groups of µSBS analysis. (b) Characterization of the dentin etch pattern and collagen layer created by the PAs.

**Table 5:** Dentin surface loss, surface roughness of the reference area (eroded surface) and of the etched area (eroded surface and PA application) with and without removal of demineralized collagen. Thickness of degraded collagen layer and surface roughness after collagen removal (roughness collagen)

	Eroded specimens
Groups	Surface loss (µm)	Collagen layer (µm)	Roughness
Reference (Ra)	After etched (Ra)	Collagen (Ra)
**Phosphoric acid application**	**PA-EXP**	1.07 (5.28)	–	0.78 (0.10)	0.73 (0.09)	–
**PA-GSE**	–3.14 (7.46)	–	0.76 (0.06)	0.90 (0.20)	–
**PA-BLU**	4.46 (6.47)	–	0.71 (0.14)	0.67 (0.12)	–
**PA-CRA**	–1.74 (1.53)	–	0.75 (0.18)	0.72 (0.09)	–
**PA-GRE**	–3.14 (3.49)	–	0.70 (0.11)	0.66 (0.13)	–
**PA-COM**	0.52 (1.96)	–	0.82 (0.15)	0.76 (0.09)	–
	Groups	Surface loss (µm)	Collagen layer (µm)	Roughness
Reference (Ra)	After etched (Ra)	Collagen (Ra)
**Phosphoric acid application and collagen removal**	**PA-EXP**	1.92 (6.26)	–0.43 (8.36)	0.79 (0.11)	0.79 (0.17)	1.20 (0.21)
**PA-GSE**	–6.77 (5.28)	0.33 (7.79)	0.78 (0.24)	0.84 (0.11)	1.12 (0.13)
**PA-BLU**	1.09 (5.91)	0.33 (10.47)	0.78 (0.16)	0.72 (0.13)	1.16 (0.23)
**PA-CRA**	0.36 (1.60)	–1.20 (9.21)	0.81 (0.19)	0.84 (0.17)	1.09 (0.21)
**PA-GRE**	3.86 (4.67)	–0.20 (8.23)	0.72 (0.14)	0.65 (0.08)	0.91 (0.20)
**PA-COM**	–4.43 (4.89)	–1.28 (6.25)	0.94 (0.24)	0.78 (0.13)	1.12 (0.23)



